# Effects of sedation on subjective perception of pain intensity and autonomic nervous responses to pain: A preliminary study

**DOI:** 10.1371/journal.pone.0183635

**Published:** 2017-09-07

**Authors:** Hongling Kang, Aya Nakae, Hiroshi Ito, Piyasak Vitayaburananont, Takehiro Minamoto, Takashi Ikeda, Mariko Osaka, Takashi Mashimo, Yuji Fujino, Satoshi Hagihira

**Affiliations:** 1 Department of Anesthesiology and Intensive Care, Osaka University Graduate School of Medicine, Suita, Osaka, Japan; 2 Graduate School of Human Sciences, Osaka University, Suita, Osaka, Japan; University of Medicine & Dentistry of NJ—New Jersey Medical School, UNITED STATES

## Abstract

Rather than relying solely on subjective pain evaluation using means such as the visual analogue scale (VAS), in clinical situations it is possible to observe evoked responses of the autonomic nervous system (ANS) as objective indicators. Few studies, however, have reported these relationships under finely controlled sedation. 16 healthy male participants were administrated in intravenous sedation with either propofol or midazolam randomly. We initially determined, using pharmacokinetic simulation, the effect-site concentration (Ce) of anaesthetic at loss of response to verbal command and eyelash reflex (Ce-LOR). Then subsequently adjusted Ce to 75%, 50%, and 25% of Ce-LOR to achieve deep, moderate, and light sedation. At awake control state and each sedation level, a noxious electrical stimulation was applied three times at the right forearm, an average pain intensity of the three stimuli was rated on a VAS (0–10). Changes in the peripheral perfusion index measured by oximetry were used as an indicator of ANS response. We analyzed the influence of sedation level on VAS and ANS responses compared to the awake control state. While ANS responses were similar in all conditions, VAS was statistically significantly lower in moderate (5.6±0.6, p <0.005) or deep (5.3±0.6, p <0.001) sedation than in the awake state (7.2±0.4). This study revealed that even when the ANS responds similarly to the same stimulation, subjective pain perception is attenuated by sedation. A cerebral mechanism other than that of the brainstem might determine subjective pain intensity.

## Introduction

Intravenous sedation is widely performed in various clinical procedures both to reduce anxiety and pain and to ensure that the patient has no awareness or memory of the procedure. There is no consensus on analgesic strategy in sedation owing to disagreement about how to assess pain during decreased consciousness. Subjective pain intensity (SPI) evaluated using a visual analog scale (VAS, 0–10) is commonly used for assessing alert-state pain. During sedation or anaesthesia, however, patients are unable to express pain perception in real time, consequently autonomic nervous system (ANS) responses, such as changes in heart rate, blood pressure, and peripheral perfusion [1–4], are commonly used as surrogate indicators. Peripheral perfusion index (PI), analyzed by pulse oximetry, is reported as an effective indicator of sympathetic nervous activity in anesthesia procedure [5] or in experimental pain study with alert consciousness [6]. These autonomic responses are useful both because they are objective measures and because their changes correlate well with alert-state SPI scores. Few studies, however, have investigated correlations between changes in SPI and ANS in sedated subjects.

In addition, it is widely held that anaesthetics do not have anti-nociceptive effects. Against this, after eliciting lower VAS pain scores from sedated subjects, Faymonville et al. have suggested that subjective pain might be attenuated by sedation [7]. Since they used anaesthetic agents combined with analgesic drugs, it is still unclear whether SPI can be reduced by anaesthetics in the absence of analgesics.

As previously mentioned, to investigate pain during sedation, it is essential to investigate how pain is perceived in not-yet-unconscious states that have been adjusted by administration of anaesthetics. Owing to large inter-individual variation in sensitivity to intravenous anaesthetics [8], there is no accepted system of monitoring functional levels of consciousness. It is possible to calculate the effect-site concentration (Ce) of anaesthetic agents by pharmacokinetic simulation [9, 10]. Ce is a virtual value which represents the drug effect as a concentration. Using such simulation, for individuals we can ascertain the Ce of anaesthetic at the point where there is loss of response to verbal command and eyelash reflex (Ce-LOR). Although Ce-LORs doses vary among individuals, it is possible to manage the sedation level of each individual by applying standardized Ce-LOR values. Thus we can finely control sedation level using pharmacokinetic simulation and clinical observation.

In this study, to investigate how SPI scores and observed ANS responses to pain vary under sedation according to standardized effects of anaesthetics, we strictly controlled levels of individual sedation by administering anaesthetic doses according to pharmacokinetic simulation; at different levels of sedation, we applied noxious electrical stimulation and compared VAS scores and PI results obtained by oximetry.

## Methods

This study was approved by the Research Ethics Committee of the Osaka University Hospital (Registration No. 11257). The current study was a part of our several researches to investigate the effect of sedation on memory or perception of several kinds of sensory inputs and tried to develop a monitoring method to prevent anesthetic awareness. At the beginning, one of our study members prepared to register our study on JMACCT (Japan Medical Association, Center for Clinical Trials), and some information were registered. We took it for granted that the registry data were properly updated by our study member. It was our fault that we started our study without checking the completion of registry. We found that it was not completed and the trial number was not assigned after finished the study. We found that it was not completed and the trial number was not assigned after finished the study. We registered our study protocols to UMIN-CTR (http://www.umin.ac.jp/ctr/, UMIN000025403), retrospectively. The authors confirm that all ongoing and related trials for this drug/intervention are registered.

**Fig 1:**
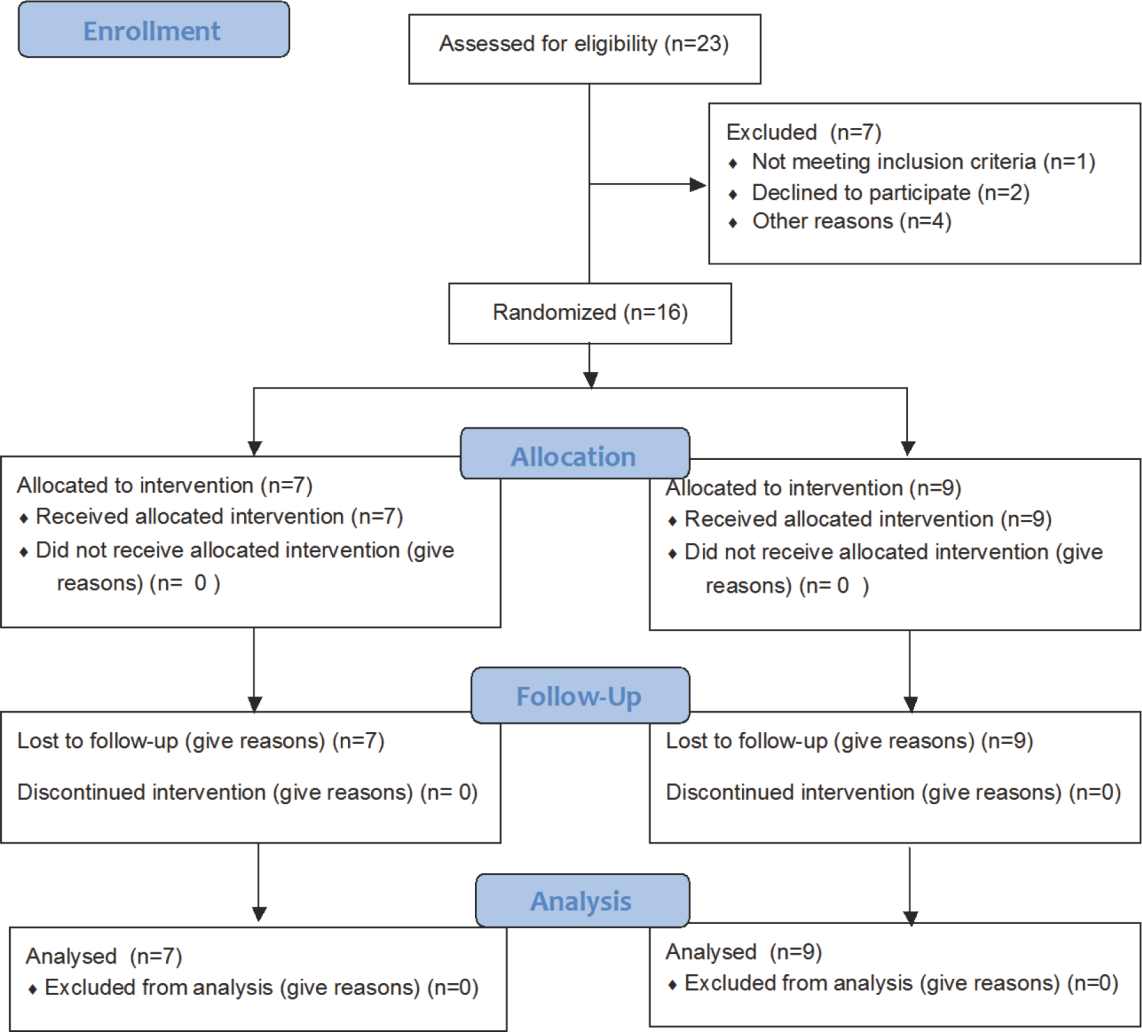
CONSORT flow diagram for the current study. 23 candidates were assessed eligibility. Finally, 16 participants were included (propofol = 7, midazolam = 9). All participants completed the study.

Participants were recruited from 1^st^ May 2013 to 1^st^ Mar 2014. We assessed for eligibility in 23 candidates. 1 candidate was excluded because of incomplete health check. 2 participants were declined before the main study because they caught cold. 4 participants were excluded in this pain perception study because of the equipment problems. Finally,16 healthy male participants were included in this study provided written informed consent (Fig 1).

### Sedation protocol

To achieve sedation, participants were administrated using either propofol or midazolam randomly (n = 7 and n = 9 respectively). Both are widely applied GABA_A_ potentiated anaesthetic agents in general use for anaesthesia or sedation procedures. Participants fasted for at least 8 h before undergoing sedation. During the procedures, they lay in bed and underwent standard anaesthetic monitoring: noninvasive blood pressure (BP), oxygen saturation (SpO_2_), and electrocardiography (ECG). Conscious level was assessed according to the Observer’s Assessment of Alertness/Sedation (OAA/S) score, and Bispectral Index (BIS; BIS-XP monitor, Medtronic, Minneapolis, USA), which is an electroencephalogram-derived measure (0–100) to monitor awareness status in anaesthesia [11]. BIS values were used when the signal quality index (SQI) was greater than 0.8. To further assess level of consciousness, we also calculated averaged BIS values for 5 min periods before the application of pain stimuli.

Before starting sedation procedure, as awakening control state, participants were required to keep quiet with alert consciousness. For each volunteer, an intravenous catheter was placed at the left forearm. Either propofol or midazolam was infused. Propofol was administered using a target-controlled infusion pump (Terufusion Syringe Pump TE-371, Terumo Co., Japan), which maintains target blood concentration constant based on population pharmacokinetics [10]. This pump calculates the assumed Ce of propofol. Midazolam was administered using a standard infusion pump (Terufusion Syringe Pump TE-332S, Terumo Co., Japan). Infusion speed was adjusted according to pharmacokinetic results yielded by TIVAtrainer software (Ver.8, EuroSIVA, http://www.eurosiva.eu/tivatrainer/TTweb/TTinfo.html), which can estimate both Ce and blood concentration of drugs.

**Fig 2:**
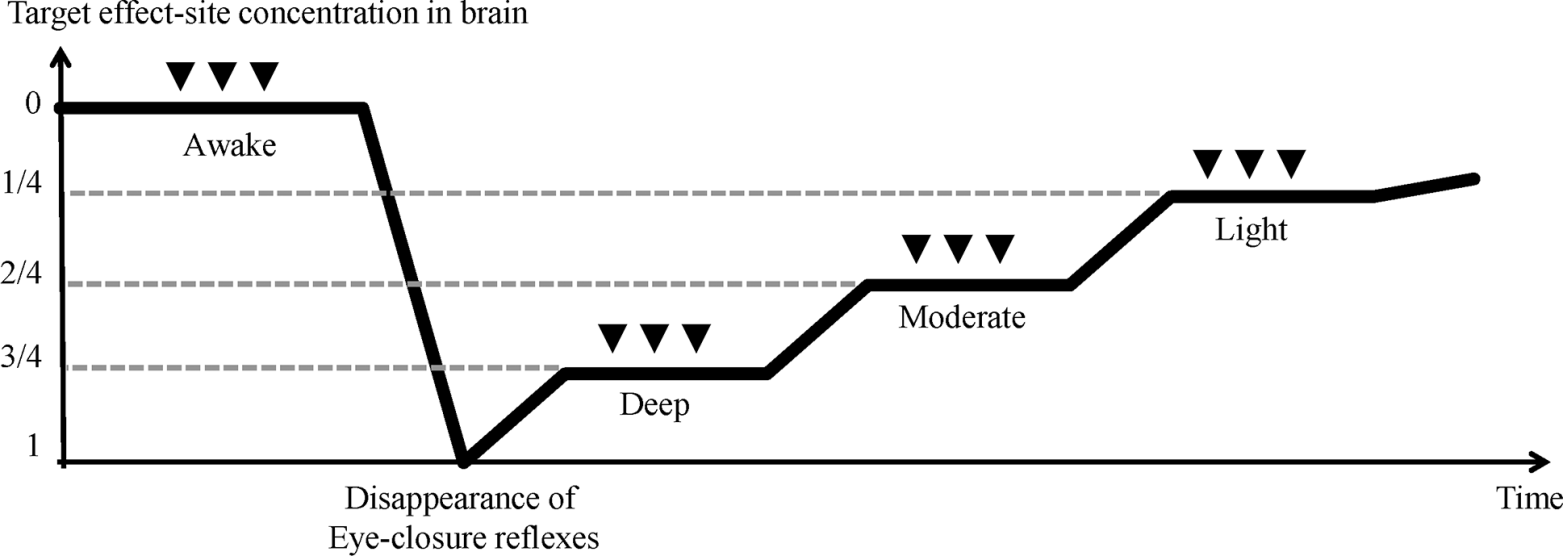
Study design of sedation procedure and electrical stimuli. The *Y* axis represents concept of target effect-site concentration (Ce). The *X* axis represents the timeline. After the awake state, sedation was induced based on drug Ce at loss of response to verbal command and eyelash reflex (Ce-LOR). Then subsequently adjusted Ce to 75%, 50%, and 25% of Ce-LOR to achieve deep, moderate, and light sedation. Inverted triangles indicate electrical stimulation, which were repeated three times in each state.

Initially, we gradually increased the drug Ce for each participant. Loss of consciousness was held to be the point of loss of response to verbal commands and lack of eyelash reflex (OAA/S score of 1). We defined this Ce as Ce-LOR (Ce at loss of response). After this, to achieve deep, moderate and light depth of sedation, drug Ce was subsequently adjusted to 75%, 50% and 25% of Ce-LOR. At each level of sedation, we adjusted the infusion pump to constantly maintain target Ce during the procedure of experimental pain. Noxious electrical stimulation was applied 5 min after drug Ce reached equilibrium. (Fig 2)

### Procedure of experimental pain

**Fig 3:**
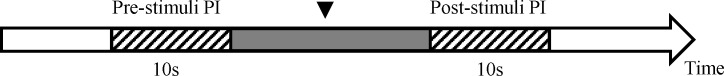
PI recording procedure for each state. Grey box, with inverted triangle above, represents a single stimulation period. The hatched boxes represent PI values recorded during 10 s periods before and after stimulation. Interval period between stimuli was 90 s. Abbreviation: Ce, effect-site concentration; PI, perfusion index; PI 1, pre-stimulus PI; PI 2, post-stimulus PI.

Noxious electrical stimulation was applied at the right forearm using the Pain Vision^®^ system (PS-2100, Nipro Co., Osaka, Japan), a device to enable quantitative measurement of pain by gradually increasing current (range 0–256 μA) [12]. First, while the volunteer was alert, we recorded pain tolerance threshold (PTT): Each was asked to press a stop button when it was felt that greater stimulation would be unendurable. If the volunteer did not press the button, PTT was recorded as 256 μA. PTT was measured three times with a 90 seconds interval period. Then the mean value for PTT was applied three times during each level of sedation (Figs 2 and 3). After the stimuli applications in each condition, volunteers were asked to score average pain intensity on the VAS.

Meanwhile, the responses of the ANS during the electrical noxious stimulation were characterized by pulse-oximetry monitoring of PI (Radical-7; Masimo, Tokyo, Japan) from the right finger. At each measurement, 10 s periods of PI before and after the application of a single stimulus (pre-stimulus PI and post-stimulus PI) were recorded. In addition, for each state, the first pre-stimulus PI value (PI baseline) was accepted as indicating the ANS activity baseline (Fig 3). Percentage changes in PI (PI% change) of pre- and post-stimulus values were calculated to assess how ANS changed at the four conscious levels (PI% change = (PI _post-stimuli_—PI _pre-stimuli_) / PI _pre-stimuli_ × 100). Both PI and BIS data were downloaded to and stored on a computer.

### Data analysis

We compared VAS scores, PI% change, and BIS values at four levels of consciousness: awake, and light, moderate, and deep sedation. We applied Shapiro-Wilk test to check whether the variables were normally distributed. To evaluate how drug type, sedation level, and sedation×drug interaction affected pain results, data were analyzed by linear mixed model (LMM) for random and fixed effects. Drug type and sedation level were considered as fixed effects, and all repeatedly measured items as random effects. We also set drug type, sedation level, or no variable, as covariant. The best LMM was chosen by the smallest Akaike’s information criterion (AIC). In post-hoc testing, the Dunnett multiple comparison test was applied to examine differences compared with alert-state results, and paired t-tests with Holm’s correction were applied to examine dose-dependent effects. Physical characteristics of the volunteers were analyzed by Student’s t-test. Statistical analysis was performed with R [13] (version 3.2.4, R Foundation for Statistical Computing, Vienna, Austria). A p-value of less than 0.05 was considered to be statistically significant.

## Results

Table 1 shows the demographic data of the participants. Table 2 shows estimated Ce of propofol and midazolam at each level of sedation. Shapiro-Wilk test revealed that all variables handled here were normally distributed. Data are presented as mean ± standard error of the mean (mean±SEM).

**Table 1:** Demographics of sedation pain-test volunteers.

Group	Sample size	Age	Weight (kg)	Height (cm)
Propofol	7	23.4±1.7	66.2±2.8	171.1±1.4
Midazolam	9	22.4±0.4	62.4±2.1	173.6±1.7

Data are represented as mean ± standard error of the mean.

**Table 2:** Effect-site concentrations (Ce) at each level of sedation.

Group	Deep	Moderate	Light
Propofol (μg ml^–1^)	1.48±0.14	1.0±0.1	0.5±0.05
Midazolam (ng ml^–1^)	30.5±3.21	20.5±2.06	10.25±1.03

Data are represented as mean ± standard error of the mean.

While no statistically significant drug-type effects or sedation×drug interactions were apparent, LMM analysis did reveal sedation effects on VAS scores and BIS values.

**Fig 4:**
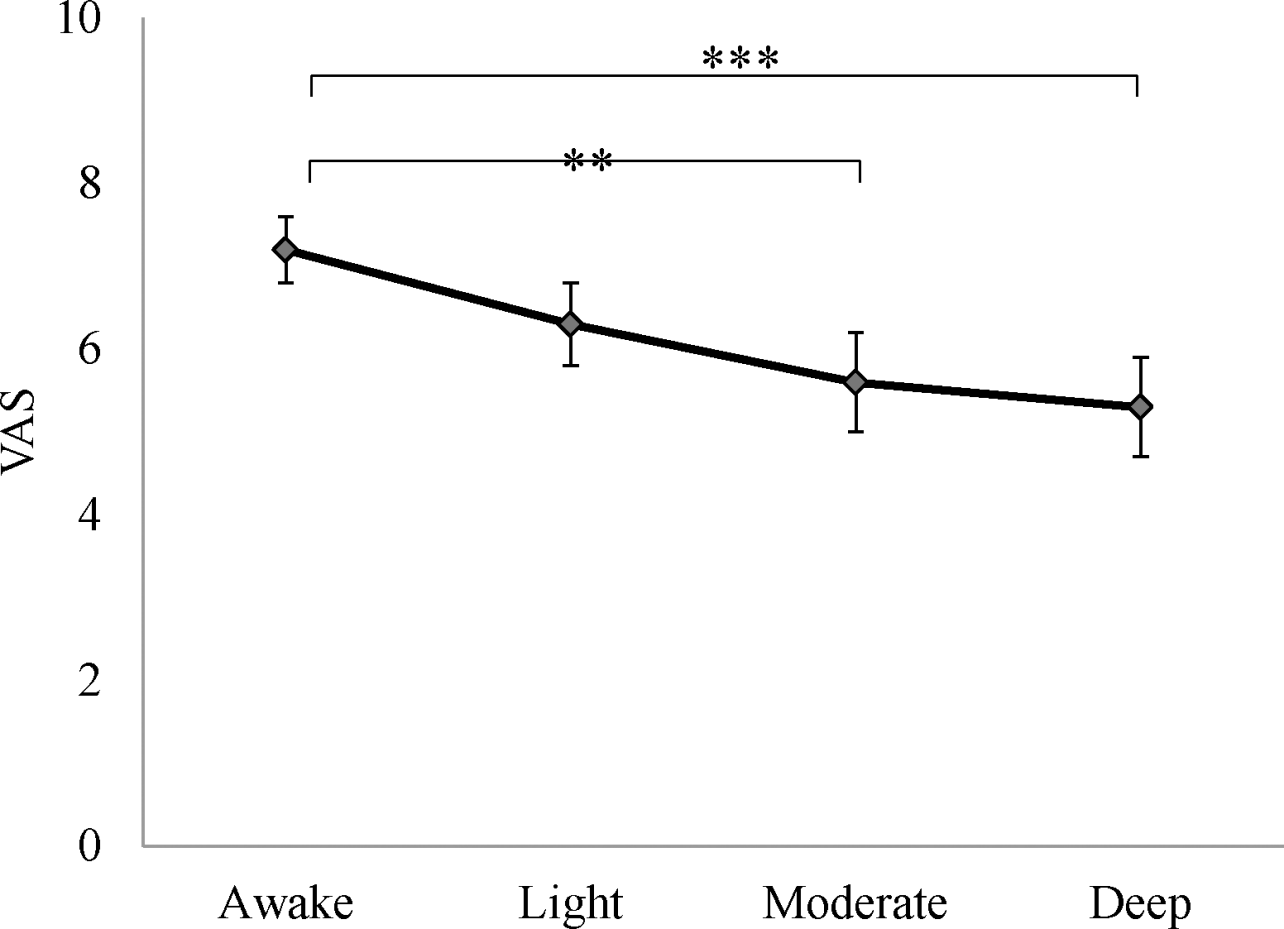
Subjective perception of pain intensity in each condition (n = 16). Error bars represent standard error of the mean. *** P<0.001; ** P<0.005. Abbreviation: VAS, visual analog scale.

**Fig 5:**
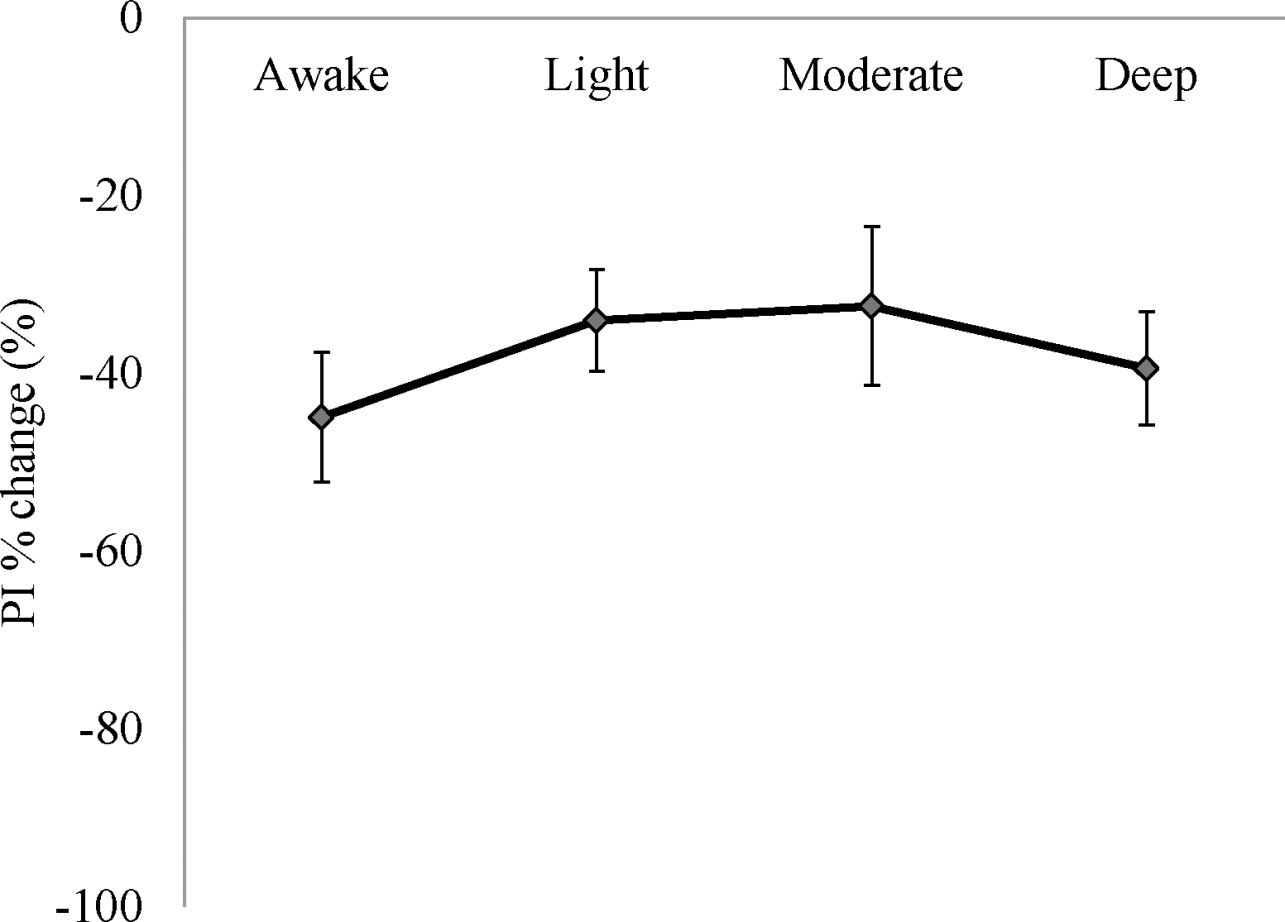
PI percentage changes before and after stimulation (n = 16). Error bars represent standard error of the mean.

Compared with the awake state, VAS scores in deep and moderate sedation were statistically significantly lower (deep vs. awake: 5.3±0.6 vs. 7.2±0.4, p <0.001; moderate vs. awake: 5.6±0.6 vs. 7.2±0.4, p <0.005) (Fig 4). PI% change would have indicated changes in ANS response during the stimulation but, when comparing results between any depth of sedation or awake state, no statistically significant differences were found in ANS values (Fig 5).

**Fig 6:**
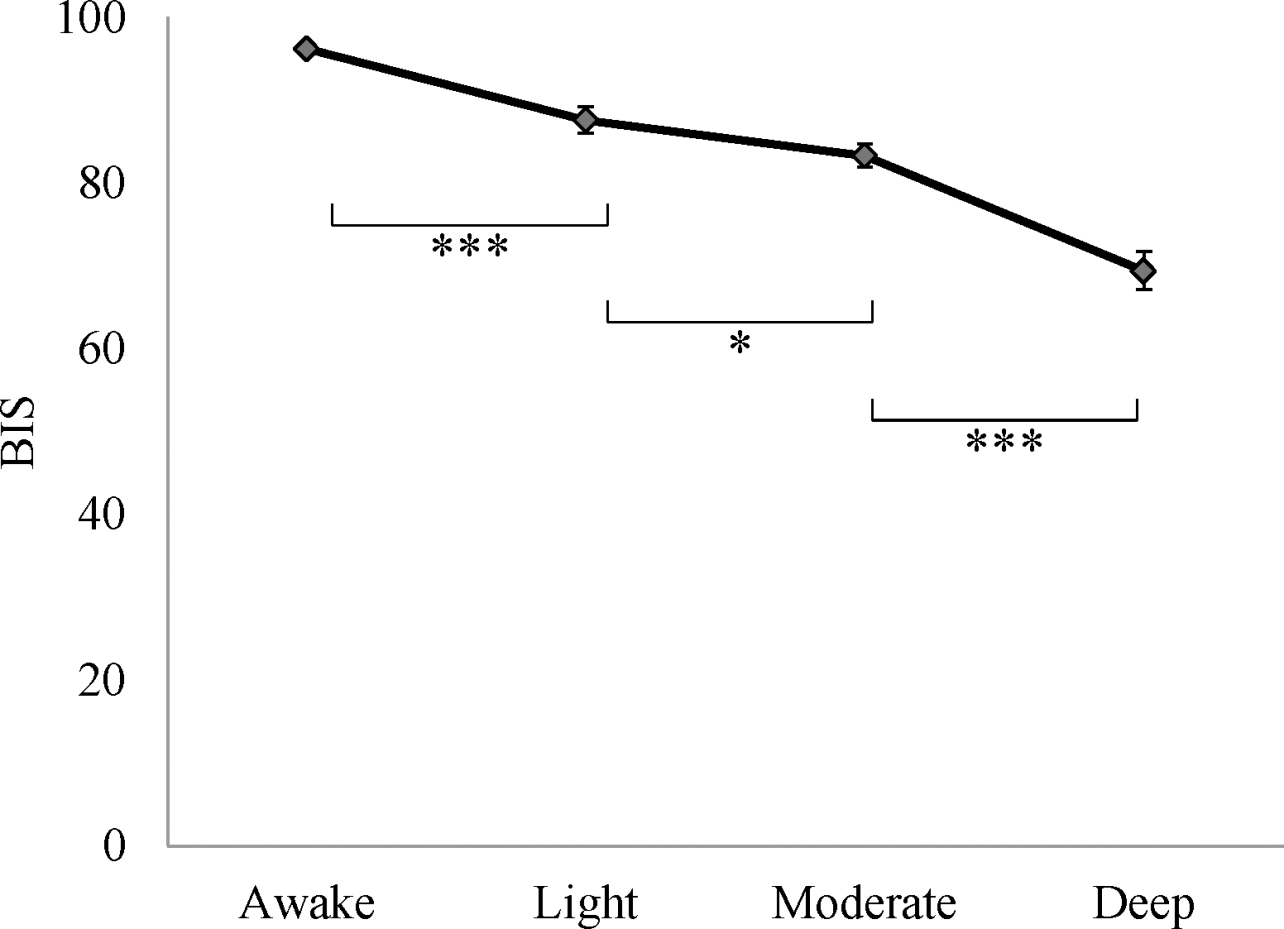
BIS values at each level of sedation (n = 16). Error bars represent standard error of the mean. *** P <0.001; * P <0.05. Abbreviation: BIS, bispectral index score.

Fluctuations in real-time BIS values during sedation procedures were observed, but the average BIS scores during the 5 min periods before stimulation statistically significantly decreased in a dose dependent manner (light vs. awake: 87.6±1.6 vs. 96.2±0.5, p <0.001; moderate vs. light: 83.3±1.4 vs.87.6±1.6, p <0.05; deep vs. moderate: 69.4±2.3 vs.83.3±1.4, p <0.001) (Fig 6). This evidence suggests that the method for controlling the level of sedation was effective.

## Discussion

We investigated how sedation affected perceived pain intensity (VAS scores) and ANS changes (PI%) when noxious stimulation was applied; we found that while VAS scores declined under sedation with propofol or midazolam, PI% remained at the same level. Pain perception modulated by the brain depends on multiple complex factors [14–16]. The decoupled changes in VAS scores and PI% in our study imply that signals from the spinal cord or in the brainstem are mediated by higher order processes that determine subjective perception of pain intensity. This insight has important implications for more effective optimization of analgesic strategies during sedation or anaesthesia.

The lower VAS scores we found during sedation, especially in deep and moderate sedation, are evidence that subjective pain relief is obtained when consciousness is decreased during sedation. By contrast, while PI decreased after stimuli, comparing the four different levels of consciousness, no statistically significant differences in the amount of reduction were found. The similarity of before-and-after changes in PI at each level of consciousness reveal a constant sympathetic nervous response to nociceptive stimuli, a response that is unaffected by sedation. In other words, regardless of the level of sedation, noxious stimuli are relayed to the brainstem with the same intensity. These results are similar to a previous study that found unchanging hemodynamic reactions to noxious stimuli during isoflurane-induced anaesthesia [17].

It is already possible to evaluate nociceptive responses in states other than waking, for example, based on observation of ANS responses or brain signals, during general anaesthesia [18–20]. But the effects of sedation on subjective pain perception remain controversial. For propofol-induced sedation, some studies have shown increased subjective pain ratings [21–24]. Other studies, including this one, present contrary findings. In healthy humans, propofol has been shown to have short-lasting analgesic effects on pain caused by intracutaneous electrical stimulation [25]. Similar pain relief was also observed at high-dose sedation [26]. In one positron emission tomography study to investigate the effects of anaesthetic on pain-evoked central processing, during sedation with low doses of propofol, a hyperalgesic effect suggested a supraspinal involvement; more interestingly, the deeper the sedation, the lower were the subjective pain ratings [27]. During sedation with midazolam, hyperalgesia effects were observed when four types of experimental pain–electrical, heat, cold, and ischemic–were applied [24]. Meanwhile, midazolam has also been shown to alleviate subjective sensations of facial pain [28]. Although contrary findings also exist, a number of previous series have demonstrated drug-specific, stimulation type-specific, and even dose-dependent effects on pain perception during sedation with anaesthetic agents. Given this growing evidence, it is perhaps necessary to look at the methodologies that have produced discrepant evidence.

In the present study, rather than eliciting initial sensation of pain or applying a constant fixed amount of stimulation across the population as was done in some studies, believing the endurable pain threshold to be an important marker for detecting analgesia or hyperalgesia [22], we started with the intensity of pain tolerance of individuals.

In addition, we found that the baseline of PI in propofol-induced sedation increased during deep sedation relative to the waking state or light sedation. For judging the onset of epidural anaesthesia, PI is known to be a sensitive indicator of inhibited activity of the sympathetic nervous system [5]. Propofol can also cause peripheral vasodilation by attenuating sympathetic nerve activity [29, 30]. Consequently, to indicate the ANS response to noxious stimulation percentage changes in PI before and after stimulation was applied.

In managing sedation, we paid attention to inter-individual variations in sensitivity to intravenous anaesthetics, variations that involve both pharmacokinetics and pharmacodynamics. For intravenous anaesthetics, at least, differences in the pharmacodynamics are thought to be the main cause of inter-individual variation. For propofol, Swinhoe et al. have shown the standard deviation of propofol blood concentration to be 0.7 μg/mL when target concentration is set at 3.5 μg/mL [9]. Although such errors have been reported, propofol infusion based on pharmacokinetic values has been proven to be safe and is widely used in daily clinical practice. In our protocols, this infusion technique enabled us to maintain a nearly constant blood and effect-site concentration of anaesthetic during maintenance of anaesthesia. By contrast, Irwin et al. reported that Ce-LOR varied from 1.7 μg/mL to 4.5 μg/mL in patients aged 18–60 years [8]. In our study, it was possible to maintain anaesthetic Ce at a constant level by adjusting the infusion pump according to pharmacokinetic simulation by TIVA trainer software. This technique enables researchers to more precisely investigate the relationship between the concentrations of anaesthetic agents and their effects.

Moreover, BIS values calculated from EEG signals have been shown to correlate well with level of consciousness [31]. Even so, fluctuations may be observed owing to contamination with artifacts such as high electromyographic activity (EMG), which tends to worsen the signal quality index [32, 33]. To minimize these problems, we were informed by BIS values least affected by artifacts. In this study, for both groups, averaged BIS values progressively decreased with the declining consciousness from awakening to deep sedation. Moreover, despite fluctuations, the standard error for BIS values in each group, even at each sedation level, was quite small. This increases our confidence that we precisely controlled consciousness by adjusting sedation, and the methodology for managing sedation used in the current study is superior to conventional methods, such as administering fixed doses of drugs.

This study is limited by its small population and single type of experimental pain stimulation. Although the effect of sedation on VAS was statistically significant, statistical power didn’t seem to be enough because of the small population. Further research using multiple models of experimental pain in a larger population is required to confirm our findings. Increasing attention is being paid to the central processing of propofol or midazolam, which have been found to reduce consciousness by disrupting whole brain integration and specific brain networks [34–37]. The investigation of cerebral mechanisms of subjective pain perception under sedation seems a promising avenue for further research.

## Conclusion

In conclusion, when sedation induced with propofol or midazolam was increased from moderate to deep, we found decreased subjective perception of pain intensity, but constant ANS responses to noxious stimulation. This revealed that the two anaesthetics have analgesic but not anti-nociceptive effects. By demonstrating that anaesthetic agents can cause subjective pain perception to go out of step with ANS responses, we have shown that level of consciousness, affected by sedation, is relevant when evaluating subjective intensity of pain perception. This suggests that cerebral processes, rather than mechanisms in the brainstem or spinal cord, are likely to determine subjective perception of pain intensity. The findings and methods of this study may help to inform clinical practice, for example, when optimizing analgesic strategies for patients undergoing intravenous sedation during medical procedures.

## Supporting information

S1 Data S1_Data of the study. (DOCX)

S1 File CONSORT checklist. (PDF)

S2 File Trial protocol as approved by the ethics committee. (DOCX)
