# Supplementary material for: Effects of sedation on subjective perception of pain intensity and autonomic nervous responses to pain: A preliminary study
Source: PLoS One. 2017 Sep 7;12(9):e0183635. doi: 10.1371/journal.pone.0183635 (PMC5589124; doi:10.1371/journal.pone.0183635)
Supplement: S1 Data — (DOCX) [file pone.0183635.s001.docx]

Supporting Information

S1 Data of the study

| Subject | Drug | Sedation | PI_pre | PI_post | %PI_diff | BIS | VAS |
| --- | --- | --- | --- | --- | --- | --- | --- |
| 6 | Midazolam | Awake | 9.52 | 4.08 | -57.14285714 | 93.8 | 7.9 |
| 6 | Midazolam | Deep | 13 | 3.52 | -72.92307692 | 74 | 5.4 |
| 6 | Midazolam | Moderate | 11.2 | 3.48 | -68.92857143 | 86.6 | 8 |
| 6 | Midazolam | Light | 6.38 | 1.84 | -71.15987461 | 93 | 7 |
| 7 | Midazolam | Awake | 1.92 | 1.48 | -22.91666667 | 94.6 | 8.2 |
| 7 | Midazolam | Deep | 2.68 | 3.02 | 12.68656716 | 76.1 | 7.3 |
| 7 | Midazolam | Moderate | 1.2 | 1.82 | 51.66666667 | 80 | 6.6 |
| 7 | Midazolam | Light | 2 | 1.92 | -4 | 82.3 | 7.95 |
| 8 | Midazolam | Awake | 1.98 | 2.7 | 36.36363636 | 95.4 | 6.4 |
| 8 | Midazolam | Deep | 9.18 | 2.6 | -71.67755991 | 71.4 | 5.4 |
| 8 | Midazolam | Moderate | 6.84 | 6.4 | -6.432748538 | 85.2 | 5.4 |
| 8 | Midazolam | Light | 11 | 2.78 | -74.72727273 | 80.5 | 8.1 |
| 9 | Midazolam | Awake | 1.8 | 1.44 | -20 | 94.2 | 7.9 |
| 9 | Midazolam | Deep | 3.18 | 1.4 | -55.97484277 | 57 | 5.4 |
| 9 | Midazolam | Moderate | 1.9 | 0.9 | -52.63157895 | 88.3 | 7.6 |
| 9 | Midazolam | Light | 0.86 | 0.56 | -34.88372093 | 95.1 | 6.3 |
| 11 | Midazolam | Awake | 10.14 | 1.98 | -80.47337278 | 97.7 | 7.9 |
| 11 | Midazolam | Deep | 5.5 | 2.52 | -54.18181818 | 61.1 | 8.5 |
| 11 | Midazolam | Moderate | 5.62 | 1.06 | -81.13879004 | 82.3 | 5.2 |
| 11 | Midazolam | Light | 1.66 | 1.78 | 7.228915663 | 81.9 | 7.9 |
| 12 | Propofol | Awake | 7.22 | 2.86 | -60.38781163 | 98 | 9.8 |
| 12 | Propofol | Deep | 6.14 | 4.44 | -27.68729642 | 69 | 9.6 |
| 12 | Propofol | Moderate | 9.14 | 3.62 | -60.39387309 | 81.2 | 9.7 |
| 12 | Propofol | Light | 9.88 | 4.1 | -58.50202429 | 84.4 | 9.7 |
| 14 | Propofol | Awake | 5.74 | 3.78 | -34.14634146 | 96.8 | 7.9 |
| 14 | Propofol | Deep | 7.98 | 4.37 | -45.23809524 | 74 | 2.3 |
| 14 | Propofol | Moderate | 9.4 | 6.78 | -27.87234043 | 93.5 | 7.6 |
| 14 | Propofol | Light | 7.22 | 5.06 | -29.91689751 | 91.7 | 7.7 |
| 15 | Propofol | Awake | 1.42 | 1.26 | -11.26760563 | 96.2 | 7.1 |
| 15 | Propofol | Deep | 9.56 | 6.08 | -36.40167364 | 78.5 | 8.6 |
| 15 | Propofol | Moderate | 1.54 | 1.04 | -32.46753247 | 86.3 | 6.8 |
| 15 | Propofol | Light | 1.6 | 1.2 | -25 | 86.4 | 8.9 |
| 16 | Midazolam | Awake | 4.84 | 2.1 | -56.61157025 | 95.3 | 3.1 |
| 16 | Midazolam | Deep | 6.16 | 2.76 | -55.19480519 | 66.3 | 2.9 |
| 16 | Midazolam | Moderate | 9.02 | 4.4 | -51.2195122 | 76.5 | 4.6 |
| 16 | Midazolam | Light | 9.68 | 7.34 | -24.17355372 | 78.2 | 4.5 |
| 17 | Propofol | Awake | 2.74 | 1.19 | -56.56934307 | 92.1 | 5.7 |
| 17 | Propofol | Deep | 8.04 | 7.46 | -7.213930348 | 73.5 | 3 |
| 17 | Propofol | Moderate | 2.4 | 1.7 | -29.16666667 | 80.3 | 3.9 |
| 17 | Propofol | Light | 2.1 | 1.52 | -27.61904762 | 94.5 | 5 |
| 18 | Midazolam | Awake | 3.88 | 2.24 | -42.26804124 | 97.9 | 8 |
| 18 | Midazolam | Deep | 3.76 | 3 | -20.21276596 | 76.2 | 5.8 |
| 18 | Midazolam | Moderate | 4.46 | 2.34 | -47.53363229 | 87.5 | 2.5 |
| 18 | Midazolam | Light | 7.24 | 4.44 | -38.67403315 | 82.9 | 3.4 |
| 19 | Propofol | Awake | 6.18 | 2.36 | -61.81229773 | 97.4 | 8.1 |
| 19 | Propofol | Deep | 14 | 5.8 | -58.57142857 | 83.5 | 4.4 |
| 19 | Propofol | Moderate | 5.08 | 1.9 | -62.5984252 | 83.6 | 5.8 |
| 19 | Propofol | Light | 1.2 | 0.84 | -30 | 95.3 | 5.5 |
| 20 | Propofol | Awake | 3.6 | 1.68 | -53.33333333 | 97.7 | 8.7 |
| 20 | Propofol | Deep | 5.96 | 4.2 | -29.53020134 | 72.9 | 4.9 |
| 20 | Propofol | Moderate | 5.6 | 2.48 | -55.71428571 | 81.5 | 4.6 |
| 20 | Propofol | Light | 2.06 | 1.72 | -16.50485437 | 82.7 | 4.5 |
| 21 | Midazolam | Awake | 3.4 | 0.92 | -72.94117647 | 98 | 7.4 |
| 21 | Midazolam | Deep | 3.32 | 1.24 | -62.65060241 | 70 | 6.2 |
| 21 | Midazolam | Moderate | 1.52 | 1.2 | -21.05263158 | 90.3 | 6.7 |
| 21 | Midazolam | Light | 1.58 | 1.1 | -30.37974684 | 96.3 | 6.4 |
| 22 | Midazolam | Awake | 3.78 | 1.48 | -60.84656085 | 95.6 | 6.9 |
| 22 | Midazolam | Deep | 2.84 | 2.82 | -0.704225352 | 49.2 | 5.1 |
| 22 | Midazolam | Moderate | 1.2 | 1.32 | 10 | 72 | 2.7 |
| 22 | Midazolam | Light | 7.56 | 2.74 | -63.75661376 | 84.2 | 4.8 |
| 24 | Propofol | Awake | 3.44 | 1.22 | -64.53488372 | 97.7 | 4.6 |
| 24 | Propofol | Deep | 7.84 | 4.29 | -45.28061224 | 57.9 | 0 |
| 24 | Propofol | Moderate | 0.9 | 1.06 | 17.77777778 | 77.9 | 1.4 |
| 24 | Propofol | Light | 0.8 | 0.62 | -22.5 | 92.5 | 3.6 |

ID1: for simulation of study protocol, not a participant.

ID2-5: Only for main studies

ID10,13,23: dropped
