# Supplementary material for: Effects of sedation on subjective perception of pain intensity and autonomic nervous responses to pain: A preliminary study
Source: PLoS One. 2017 Sep 7;12(9):e0183635. doi: 10.1371/journal.pone.0183635 (PMC5589124; doi:10.1371/journal.pone.0183635)
Supplement: S2 File — (DOCX) [file pone.0183635.s003.docx]

Research Protocols

Research of memory during sedation in volunteers

(Original is written in Japanese)

Responsible person: Satoshi Hagihira

Associate Professor, Department of Anesthesiology and Intensive Care Medicine,

Osaka University Hospital

2-15 Yamadaoka, Suita City, Osaka, 565-0871

Tel. +81-6(6879)3133, Fax. +81-6(6879)3139

E-mail: [hagihira@anes.med.osaka-u.ac.jp](mailto:hagihira@anes.med.osaka-u.ac.jp)

12^th^, Dec 2011. First Version.
18^th^, Mar 2013. Final version.

18^th^, Mar 2013. Approved by the Research Ethics Committee of the Osaka University Hospital

　　　　　　　　　　　　List of contents

1. Overview ......................................................................................................... 5
2. Purpose ........................................................................................................... 5
3. Background .................................................................................................... 5
   2.1 Awareness during anesthesia ................................................................. 5
   2.2 Standard monitor for awareness during anesthesia ............................. 6
   2.3 Results of the previous studies ............................................................... 6
   2.4 Significance of the current study ............................................................ 7
   2.5 Predicted risk and expected benefit ........................................................ 7
4. Design of the study ........................................................................................ 8
5. Method of inclusion ........................................................................................ 8
   4.1 Inclusion criteria ...................................................................................... 8
   4.2 Exclusion criteria ..................................................................................... 8
   4.3 Number of participants ............................................................................ 8
   4.4 Way of subscription .................................................................................. 8
6. Informed consent ............................................................................................ 8
7. Registration and assignment ......................................................................... 9
8. Research protocols .......................................................................................... 9
   7.1 Method of sedation ................................................................................... 9
    7.1.1 Propofol ................................................................................................ 9
    7.1.2 Midazolam ........................................................................................... 9
   7.2 Basis of infusion rate ............................................................................... 9
   7.3 Criteria for discontinuation ..................................................................... 10
9. Safety of the participants ............................................................................... 10
10. Observation, checking items and schedule ................................................... 11
    9.1 Observation period ................................................................................... 11
    9.2 Observation, checking items .................................................................... 11
     9.2.1 Pre test ................................................................................................. 11
     9.2.2 Studies under sedation ........................................................................ 12
     9.2.3 follow-up study ..................................................................................... 13
11. Assessment and report of adverse effect ....................................................... 13
    10.1 Definition ................................................................................................ 13
     10.1.1 Definition of adverse effect .............................................................. 13
     10.1.2 Definition of serious adverse effect ................................................. 14
    10.2 Contents of recording fro the adverse effect ......................................... 14
    10.3 Determination of serious level ............................................................... 14
    10.4 Determination of Severity ...................................................................... 14
    10.5 Predicted adverse effect ......................................................................... 14
    10.6 Handling and report of adverse effect ................................................... 15
     10.6.1 Handling of the serious adverse effect ............................................ 15
     10.6.2 Other reports .................................................................................... 15
     10.6.2.1 Safety information about drugs or medical instruments ........ 15
     10.6.2.2 Report of new safety information ............................................. 15
12. Deliberations by the committee of safety and effectiveness ......................... 15
    11.1 Deliberations by the committee of safety and effectiveness ................. 15
    11.2 Regular meeting ...................................................................................... 15
    11.3 Meeting on demand ................................................................................ 15
    11.4 Admonition .............................................................................................. 15
13. Number of participants .................................................................................. 16
    12.1 Aimed number of participants, and its basis ........................................ 16
    12.2 Study Periods .......................................................................................... 16
14. Evaluation parameters ................................................................................... 16
    13.1 Primary endpoint .................................................................................... 16
    13.2 Secondary endpoint ................................................................................ 16
15. Statistics .......................................................................................................... 16
    14.1 Objective group ....................................................................................... 16
    14.2 Criteria of data handling ........................................................................ 16
     14.2.1 Safety ................................................................................................ 16
     14.2.2 Missing data ..................................................................................... 16
    14.3 Method of analysis .................................................................................. 16
     14.3.1 Safety ................................................................................................ 16
     14.3.2 Main parameters ............................................................................. 16
     14.3.3 Additional parameters .................................................................... 16
16. Data collection and management .................................................................. 16
    15.1 Data collection ........................................................................................ 16
    12.2 Protection of personal information ........................................................ 17
17. Discontinuation of the study .......................................................................... 17
18. Ethical issues .................................................................................................. 17
    17.1 Rules to respect ....................................................................................... 17
    17.2 Informed consent .................................................................................... 17
    17.3 Approval of the institute ........................................................................ 17
19. Costs of the research ......................................................... ............................ 18
    18.1 Funding and Conflict of interest ............................. ............................. 18
    18.2 Budget ..................................................................................................... 18
    18.3 Compensation for health hazard ........................................................... 18
20. Utilization and preservation of the data .................................................... 18
21. Publication of the outcomes ........................................................................... 18
22. Organization of the research.......................................................................... 18
     21.1 Responsible person ............................................................................. 18
     21.2 Head office of the study ...................................................................... 18
     21.3 Registration center ............................................................................. 19
     21.4 Responsible person for Statistics ....................................................... 19
     21.5 Responsible person for data management ......................................... 19
     21.6 Responsible person for assignment .................................................... 19
     21.7 Assessment of the Safety .................................................................... 19
23. References ........................... ........................................................................... 20
24. Appendix ... ........................... .......................................................................... 21
25. Overview

We will investigate the response to noxious stimuli, perception of the stimuli, changes of electroencephalogram (EEG), and memory performance under sedation induced by propofol or midazolam in volunteers.
As to the memory performance, we adopted explicit-memory task test, and implicit-memory task test.

1. Purpose

The main purpose of the current research is to know how to construct an anesthesia monitor to prevent awareness during anesthesia by measuring the response to noxious stimuli, perception of the stimuli, changes of electroencephalogram (EEG), and memory performance under sedation in healthy volunteers.

1. Background

2.1 Awareness during anesthesia

Now, anesthesia was composed of three elements; unconsciousness, analgesia (anti-nociception), and immobilization. Anti-nociception and immobilization are managed by analgesic such as opioids, and neuromuscular blocker. If a patient was aware during anesthesia, he/she might be unpleasant, and they might know undesirable information during surgery^1)^. Awareness during anesthesia defines the state that a patient was aware during anesthesia even when anesthesia was provided to induce unconsciousness and amnesia^2)^. Awareness during anesthesia is usually accompanied with memory recall.
Recall of intraoperative events in patients under anesthesia is rare (0.2-0.7%)^4)^, namely one or two patients per thousand cases would suffer awareness during anesthesia. Some reports showed that fairly number of patients suffered awareness during anesthesia would progress post-traumatic stress disorder (PTSD)^3)^. As the symptoms of PTSD, depression, insomnia, flashbacks, amnestia are known^3)^.
Currently, The major causes of awareness during anesthesia were considered as the following two; the first cause is the absolutely or relatively insufficient administration of anesthetic and/or analgesic, and the second cause is the failure of anesthetic and/or analgesic administration such as infusion line trouble or mistake of drug preparation^5-7)^. On the other hand, over-administration of anesthetic and/or analgesic delayed the recovery from anesthesia after surgery, and might increase the incidence of adverse effects^2)^.
Awareness during anesthesia is now supposed to be of much interest because of the following reasons; (1) lighter anesthetic level is required for quicker recovery, (2) news report about awareness during anesthesia or drama handling it is increasing, (3) anesthesiologists are more often sued for awareness during anesthesia, and median claimable amount is 34 thousand dollar (maximum was 800 thousand dollar), (4) anesthesia monitor is now widely used^4)^.

2.2 Standard monitor to prevent awareness during anesthesia.
To prevent awareness during anesthesia, we have to use brain monitor, such as EEG or evoked potential, besides the observation of hemodynamic change or movement. Currently, Bispectral index (BIS™) is widely used to assess the level of hypnosis and try to prevent awareness during anesthesia. BIS™ monitor is the only monitor that was approved as the monitor of consciousness by Food and Drug Administration, and has some advantages compared with the previous ones. Sensor of BIS™ monitor is easier to use for end-users, and it enables to get low impedance without difficulty. BIS™ monitor is known to use coefficients obtain by the multi-variate analysis of EEG database, and predicts the clinical level of hypnosis. However, incidence of awareness during anesthesia didn’t decrease even using BIS™^7,9)^. BIS seems to be effective as the monitor of hypnosis, as it uses the EEG signals obtained from the frontal cortex that is related to consciousness, but it doesn’t work as the monitor of memory, because memory is mainly controlled by hippocampus and amygdala^7)^. The manufacturer recommended to keep the BIS values between 40 to 60 during anesthesia, which could not prevent awareness during anesthesia completely, because BIS values are “predicted” value of level of hypnosis, and it cannot predict the responsibility to stimuli. BIS values, hemodynamic changes, and movements against the surgical stimuli aren’t reliable monitors for awareness during anesthesia. Thus, currently there is no effective monitoring method of memory during anesthesia, which is one of the reasons why we could not prevent memory recall after surgery.
2.3 Results of the previous studies
There are two types of memory, explicit-memory and implicit-memory, when we think about the memory during anesthesia. The essential difference between them is whether learning occurs with or without concurrent awareness of remembering. The patient could not express in words about implicit-memory, although it was actually remained in the brain. It is known that implicit-memory also has influence on the patients’ behavior after surgery^6)^. Generally, previous studies focused on the explicit-memory. Those studies used questionairing after surgery, but we can’t investigate the existence of implict-memory by this method. Some researchers investigated the existence of the implicit-memory by using hypnotic suggestion. The problem is that we could not quantify the implicit-memory by a simple method. Thus memory after surgery is underestimeted by this method. Currently, it is unclear that implicit-memory could remain at surgical level of anesthesia^6)^. It is important to investigate explicit-memory and implicit-memory, respectively.
2.4 Significance of the current study
The goal of this study is to explore the fundamental knowledge to develop a reliable monitoring system to prevent awareness during anesthesia.

Previous study showed that 37% of the patients who suffered awareness during anesthesia reported it for the first time on the day later than 7^th^ post-operative day^10)^. This suggested that we have to investigate the memory not only at just after the event, but also at a week or later. In the current study, we enroll healthy volunteers, and observe the fixation of memory under sedation. It is known that memory performance is varied by gender, age, ASA-PS (American Society of Anesthesiologists physical status), type of surgery, and medical history. So, we only include young healthy male volunteers. We use propofol or midazolam as sedative, because we are quite familiar with these drugs. Propofol is infused using target-controlled infusion (TCI) pump, and estimated effect-site concentration (Ce) is adjusted between 0.2 μg/mL to 2.5 μg/mL. Midazolam is infused using standard syringe pump with the aid of pharmacokinetic simulation software (TIVATraniner; Ver 8, EuroSIVA, <http://www.eurosiva.eu/tivatrainer/TTweb/TTinfo.html>), and Ce of midazolam is adjusted between 10 ng/mL to 60 ng/mL. In these range of Ce, both drugs are quite safe. Both drugs potentiate GABA_A_ receptor function in amygdala and suppress the memory formation and induce amnesia^14)^. Especially, midazolam causes anterograde amnesia^15,16)^. Propofol is not cause retrograde amnesia. One previous study concluded that midazolam strongly suppress the memory formation of a painting at painful stimuli than propofol^18)^, but another study revealed that the effect of both drugs were similar^19)^.
As written above, we often use both drugs and the profiles of amnestic effects are not known in detail. Then, we apply several kinds of memory task test, and clarify it. Furthermore, we simultaneously observe EEG and event related potentials to know the sedative effect of these drugs at the time of the task, and explore what level of sedation or anesthesia will be required to suppress the memory formation. We exclude the elderly persons in the current study, because their memory function may be impaired by aging or other factors.
2.5 Predicted risk and expected benefit
We administer propofol and midazolam according to the method written in the product documents, and the following adverse effects have been reported.
• Serious adverse effects; hypotension, anaphylactoid reations, bronchospasm, upper airway obstruction, seizure like movement, serious bradycardia, pump failure, ventricular tachycardia, ventricular ectopic rhythm, left bundle branch block, lung edema, delay of awakening, rhabdomyolysis, malignant hyperthermia like symptoms, anaclisis, apnea, respiratory depression, anaphylactic reaction, cardiac arrest, malignant syndrome.
• Other adverse effects; bradycardia, ST depression, headache, tremor, hiccup, cough, nausea, sialism, vomiting, pacreatitis, reddening, erythema, AST↑, ALT↑, ALP↑, LDH↑, γ-GTP↑, bilirubin↑, renal dysfunction, colored urine (white, green), euphric, difficult antaphrodisiac, delirium, WBC↑, hypoproteinemia, hypoalbuminemia, pain on injection, phlebitis, thrombosis, fever, trepidation, arrhythmia, hypotension, hypertension, tachycardia, atrial fibrillation, vertigo, CK↑, etc.

1. Design of the study
   Comparative, unblinded, prospective, single center study
2. Method of inclusion
   4.1 Inclusion criteria
   1) healthy male aged from 20 to 40 when informed consent is obtained
   2) obtained written informed consent
   3) passed the medical check-up within a year, and certified it. Medical check-up includes measurement of height, weight, blood pressure, urine test (protein, occult blood, sugar), chest x-ray, hearing test, and vision test, ASA-PS of I or II.
   4.2 Exclusion criteria
   1) with a history of allergy to propofol
   2) with a history of allergy to midazolam
   3) with a history of epilepsy
   4) with a history of drug abuse
   5) with a history of drug allergy
   6) with a history of brain diseases
   7) with a history of alcohol dependency
   8) a person who we judged as inappropriate for the study
   4.3 Number of inclusion
   Totally 20 cases.
   4.4 Way of subscription
   We recruit participants using advertisement for subscription on the web. If we can’t gather enough number of participants, we will offer public subscription according as the phase I clinical trial.
3. Informed consent
   1) Before starting the study, the attending staff must hand over the briefing paper which is approved by the research ethical committee, and explain the following contents, and obtain written informed consent.
   2) If the candidate agrees, the doctor who explains to the candidate must write the candidate’ s name, date and the doctor’s name on the consent form. The candidate also subscribes to the agreement.
   3) Research staff makes a copy of the consent form and hands over it with the briefing paper to the candidate.
4. Registration and assignment
   6.1 Registration
   1) Research staff fills the registration form and scans it, and then sends the PDF file to the registration center.
   2) Registration center checks the qualification.
   3) Registration center assigns the group, and brings out the registration card, which is kept in the Experimental psychology laboratory.
   4) Registration center informs the result of assignment by e-mail.
   5) Responsible person keeps the registration card.
   Registration center; nosaka@hus.kyoto-u.ac.jp
   6.2 Method of assignment
   Random block assignment method
5. Study protocols
   7.1 Method of sedation
   In the current study we use propofol or midazolam for sedative agent.
   An anesthesiologist administers either drug to a participant who is fulfilled the inclusion criteria and not corresponding to the exclusion criteria.
   7.1.1 Propofol
   Drug: propofol, 1% Diprivan kit® (Astra Zeneca, Inc.), propofol 10mg/ml.
   Propofol is infused using target-controlled infusion (TCI) pump, and estimated effect-site concentration (Ce) is adjusted. Increment or decrement of Ce is set about 0.3 μg/ml.
   7.1.2 Midazolam
   Drug: midazolam, Dormicum® (Astellas, Inc.), Midazolam 10mg/ml.

Midazolam is infused using standard syringe pump with the aid of pharmacokinetic simulation software (TIVATraniner; Ver 8, EuroSIVA, <http://www.eurosiva.eu/tivatrainer/TTweb/TTinfo.html>). Ce of midazolam adjusted from 10 ng/ml to 60 ng/ml.
7.2 Basis of infusion rate
• Propofol
Initial setting is determined at 2.0 μg/ml, according to the report by Barakat AR et al.^11)^. Considering the 25% of standard deviation12), we set the upper limit as 2.5 μg/ml. Actually, we sometimes experience a young patient who can speak at more than 3.0 μg/ml.
• Midazolam
As interindividual variation of sensitivity of midazolam is larger than that of propofol, we set the standard target Ce as 40 ng/ml, and we set the maximum target Ce as 60 ng/ml^13)^.
With this method, we can maintain the Ce of propofol or midazolam constant during each series of studies.
7.3 Criteria for discontinuation
If one of the following situations is confirmed by the doctor who is carrying out the study, we will stop the experiments, and execute appropriate procedures and/or treatments.
1) in case that the participant withdraws the informed consent
2) in case that violation of the inclusion or exclusion criteria is revealed
3) in case that discontinuation is judged due to the adverse effects
4) in case that drug allergy is suspected
5) in case that nausea induced by the used drug prevents the continuation of the study
6) other reasons that the doctor who is carrying out the study decide the discontinuation

1. Safety of the participants
   To confirm the safety of the participants, the doctors must comply with the following basic matters.
   (1) Follow the study protocols
   (2) Pay much attention to identify the adverse effect early, and start the appropriate procedures and/or treatments as soon as possible if the doctors identify it.
   For the participant’s safety, study is done in the operating room of Osaka University Hospital, and three anesthesiologists and a nurse manage the sedation. Anesthesiologists are well trained to administer these drugs. To manage hypotension or respiratory depression, devices for emergency are made to ready use.
   If serious adverse effect is occurred, anesthesiologists must accommodate to the following flow chart.

Flow chart for emergency


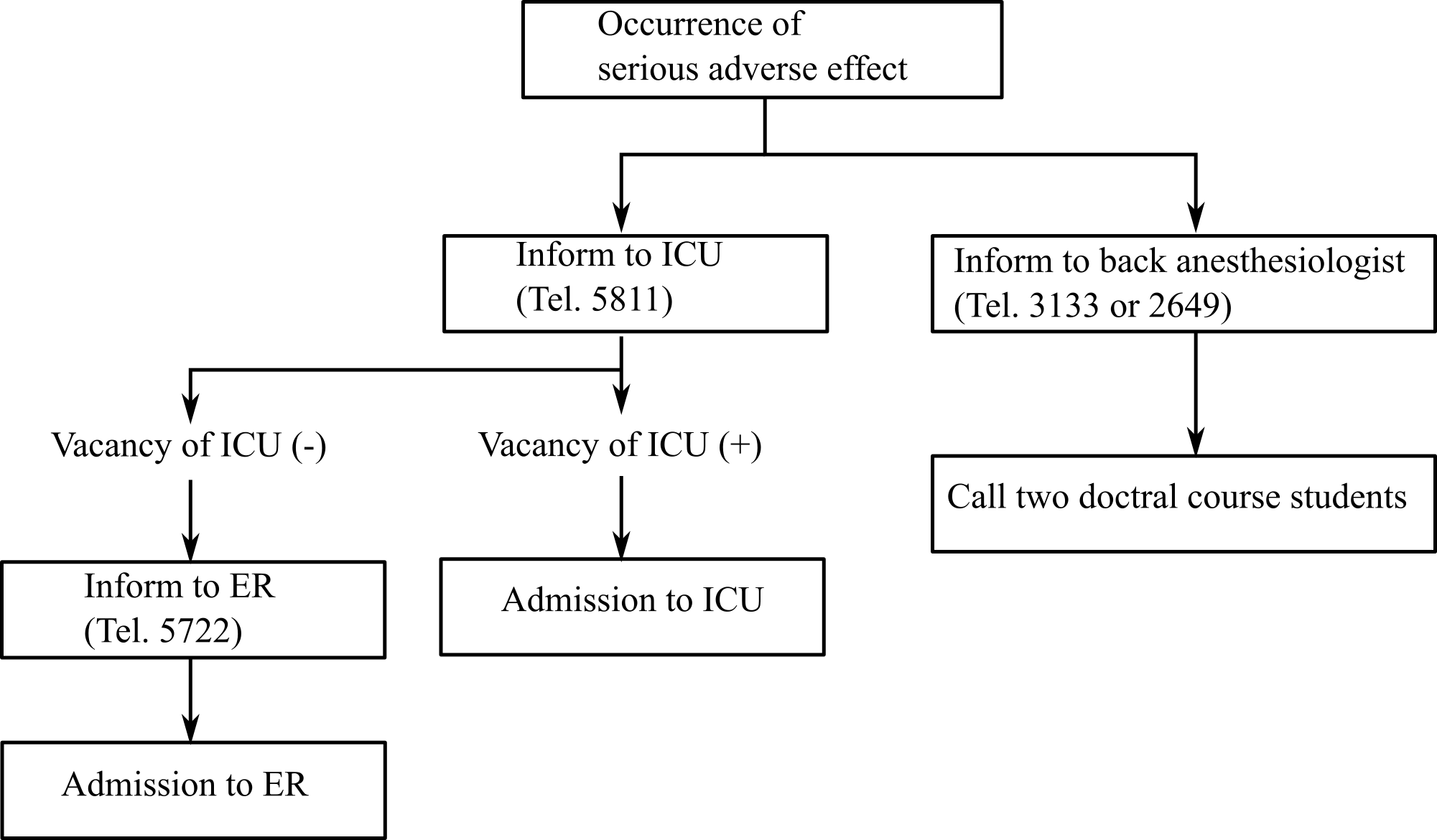


1. Observation, checking items and schedule
   9.1 Observation period
   From 1^st^ May 2013 to 31th Mar 2014 (registration is until 1st Mar 2014)
   9.2 Observation, checking items
   9.2.1 Pre test
   One week before the main study, we check and observe the following items.
   1) certification of medical check
    Height, weight, blood pressure, urine test (protein, occult blood, sugar), chest X-ray, audiometry, visual activity
   2) backgrounds of the participant
   birthday, gender, height, weight
   complications, medical histories based on interview sheet.
   3) physical examination
   Subjective: by interview sheet
   Objective: a doctor does physical examination
   4) EEG recording
   Record the raw EEG as well as EEG derived parameters using BIS™ monitor.
   5) Pain threshold test and event-related potentials (ERP)
   Confirm the pain threshold is within the normal limits. Appearance of ERP is also checked.
   6) Memory task test
   Implicit-linguistic memory task, implicit-visual memory task, explict-linguistic memory task, and explicit-visual memory task test
   9.2.2 Studies under sedation
   Sedation is provided in the operating room of Osaka University Hospital.
   Baseline data are gathered before sedation.
   At first, Ce of sedative is gradually increased until response to verbal command is lost. At this point of Ce is defined Ce-LOR (loss of response). And sedation level is controlled as deep (3/4 of Ce), moderate (1/2 of Ce), and light (1/4 of Ce). After obtained the steady state, each measurement or tasks are applied.
   1) Observations
   Conscious level (GCS)
   Respiratory function: Oximetry (SpO2, RR)
   Circulatory status: NIBP, HR, ECG
   Motor function: ability of grip hands
   Pain sensation: VAS (visual analog scale)
   2) EEG recording
   3) Pain perception test
   4) Memory task test
   Implicit-linguistic memory task, implicit-visual memory task, explict-linguistic memory task, and explicit-visual memory task test

   In each step, wait more than 5 minutes after reaching the Ce to the target level.
   After finish the all measurement, Ce is changed to the next step.

   • Finish of the study protocols
   When all physical status clear the criteria for leaving room, the participant can leave the room after 5 minutes of confirmation. (consciousness clear, activity normal, stable hemodynamics, oxygen saturation normal, no pain, no nausea or vomiting)

   9.2.3 follow-up study
   One week after the main test, we do follow-up study.
   1) Observations
   2) Memory task test
   Implicit-linguistic memory task, implicit-visual memory task, explict-linguistic memory task, and explicit-visual memory task test

|  | Pre-test | Test | | | | | Post-Test |
| --- | --- | --- | --- | --- | --- | --- | --- |
| Time course | 1 week before | At start | Sedation level 1 | Sedation level 2 | Sedation level 3 | Recovery /Canceled | 1 week after /Canceled |
| Informed consent | 🞆 |  |  |  |  |  |  |
| Checking of the participant | 🞆 |  |  |  |  |  |  |
| Sedation |  | 🞆 | 🞆 | 🞆 | 🞆 | 🞆 |  |
| Subjective and Objective Symptoms |  | 🞆 | 🞆 | 🞆 | 🞆 | 🞆 | 🞆 |
| ERP | 🞆 |  | 🞆 | 🞆 | 🞆 |  |  |
| EEG |  | 🞆 | 🞆 | 🞆 | 🞆 | 🞆 |  |
| Memory task test | 🞆 | 🞆 | 🞆 | 🞆 | 🞆 | 🞆 | 🞆 |
| Checking of the adverse effects |  | 🞆 | 🞆 | 🞆 | 🞆 | 🞆 | 🞆 |

1. Assessment and report of adverse effect
   10.1 Definition
   10.1.1 Definition of adverse effect
   Adverse effects are defined as any undesirable or unpredictable symptoms or diseases identified the participants of the study after inclusion, which are not always caused by the procedures of the study.
   When we identify the adverse effect, we describe in the hospital record, and execute appropriate procedures and/or treatments. We also report it using the reporting system of our hospital.
   10.1.2 Definition of serious adverse effect
   Serious adverse effects are defined as the adverse effect which may lead to death, is life-threatening, require admission, prolong the hospital stay, or remain permanent or serious sequelae.
   10.2 Items of recording the adverse effect
    Type of the adverse effect:
    Date occurred or identified:
    Date disappear:
    Serious level:
    Severity:
    Need of treatment:
    Outcome:
    Causal relationship:
    Treatment (if required):
   10.3 Determination of serious level
    Light: No problem exist in daily life.
    Moderate: Difficulty exists in daily life, but possible.
    Severe: Highly difficulty exists in daily life.
   10.4 Determination of Severity
    The severity of each adverse effect is judged according to its criteria.
    Severe:
    Not severe:
   10.5 Predicted adverse effect
    1% Diprivan kit® (Astra Zeneca, Inc.)
    hypotension (≥5%)
    anaphylactoid reations (<0.1%)
    bronchospasm (<0.1%)
    upper airway obstruction or transient apnea (0.1-<5%)
    seizure like movement (0.1-<5%)
    serious bradycardia (0.1-<5%), pump failure (<0.1%)
    ventricular tachycardia (<0.1%), ventricular ectopic rhythm (0.1-<5%)
    left bundle branch block (<0.1%)
    lung edema (<0.1%)
    delay of awakening (0.1-<5%)
    rhabdomyolysis (<0.1%)
    malignant hyperthermia like symptoms (<0.1%)
    Dormicum® (Astellas, Inc.)
    anaclisis (unknown)
    apnea (unknown), respiratory depression (0.1-<5%)
    anaphylactic reaction (unknown)
    cardiac arrest (unknown)
    malignant syndrome (unknown)
   10.6 Handling and report of adverse effect
    Adverse effect arises within 7 days after the study must be reported using case report form. When adverse effect is identified, start the appropriate treatment as soon as possible, and describe the medical records. If serious adverse effect is identified, send the report to the dean of the hospital.
   10.6.1 Handling of the serious adverse effect
    Responsible person must inform about the serious adverse effect to the dean of the hospital and related departments orally or by phone within 72 hours, and send fax or e-mail (primary report).
   Furthermore, submit the serious adverse effect report form to the dean of the hospital and related departments within 7 days.
   10.6.2 Other reports
   10.6.2.1 Safety information about drugs or medical instruments
    If responsible person considers that the adverse effect might spread widely, he must report it to the Ministry of Health, Labor and Welfare, Japan.
   10.6.2.2 Report of new safety information
    If we get a new safety information from the pharmaceutical company, we will report it to the dean of the hospital in a writing form.
2. Deliberations by the committee of safety and effectiveness
   11.1 Deliberations by the committee of safety and effectiveness
    The committee of safety and effectiveness deliberate the following items periodically. It is also organized on demand.
   11.2 Regular meeting
    Responsible person reports the progress of the study every year to the committee.
   11.3 Meeting on demand
    When following event is identified, meeting is held on demand.
    1) Problem is identified on the progress of the study.
    2) Adverse effect that judged to require deliberation is identified.
    3) Adverse effect is reported from the similar treatment or study.
    4) Other case that the responsible person or committee judges the requirement of deliberation.
   11.4 Admonition
    When the admonition is proposed, the committee admonish it by unanimous vote. If some of the member doesn’t agree with it, the committee admonish it with accessory comments.
3. Number of participants
   12.1 Aimed number of participants, and its basis
    Totally 20 cases (10 cases each)
   12.2 Study Period
    From 1^st^ May 2013 to 31^th^ Mar 2015
    (Registration: 1^st^ May 2013 to 1^st^ Mar 2014)
4. Evaluation parameters
   13.1 Primary endpoint
    Changes of EEG
   13.2 Secondary endpoint
    Memory task performance
    Assessment of Safety
    • Incidence of adverse effect
    • Incidence of serious adverse effect
5. Statistics
   14.1 Objective group
    All recorded data
   14.2 Criteria of data handling
    14.2.1 Safety
    Adverse effect arises within 7 days after the study
    14.2.2 Missing data
    We don’t complement the missing data. However, appropriate method can be used for statistical analysis.
   Main parameters
   14.3 Method of analysis
    14.3.1 Safety
    All adverse effects are compiled, and the number and the incidence are recorded.
    14.3.2 Main parameters
    Comparison of memory task score between the drugs is analyzed by Man-Whitney U-test. Comparison within a group is analyzed by Freidman test.
    14.3.3 Additional parameters
    Comparison of BIS values between the drugs is analyzed by Man-Whitney U-test. Comparison within a group is analyzed by Freidman test.
6. Data collection and management
   15.1 Data collection
   The following data sheets are used.

|  | leaflet | limit of submission | reporting method |
| --- | --- | --- | --- |
| 1 | interview record | at registration | e-mail |
| 2 | registration record | at registration | e-mail |
| 3 | registration verification | before sedation | e-mail |
| 4 | protocol list | at the end of main study | e-mail |
| 5 | results report | at the end of main study | e-mail |
| 6 | criteria of leaving OR | at the end of main study | - |
| 7 | criteria of return home | at the end of main study | - |

1. Discontinuation of the study
   If one of the followings is identified, responsible person must report it to the dean of the hospital and related departments.
   1) Monitoring of the study or the progressing report reveals the difficulty in continuation of the study.
   2) Safety of the study are suspected by the occurrence of serious adverse effect
   3) The newly published article or presentation of the meetings suspects the safety of the study or it cancels the significance of the study.
2. Ethical issues
   17.1 Rules to respect
   All researchers must comply with the “Declaration of Helsinki, updated 2008”, and “The guidance of ethical issues in clinical researches” published by the Ministry of Health, Labor and Welfare, Japan. Updated 2009.”
   17.2 Informed consent
    Before starting the study, the attending staff must hand over the briefing paper which is approved by the research ethical committee, and explain the following contents, and obtain written informed consent.
   1) Attendance to the study is left his/her option.
   2) No disadvantage exists if he/she doesn’t attend.
   3) Participant can withdraw the consent any time without penalty.
   4) The reason why the candidate is selected.
   5) Purpose of the study, the protocols of the study and the periods of the study
   6) Researcher’s name and title
   7) Expected results, and predictable risk or inconvenience
   8) Access to the information of the study
   9) Handling of the personal data
   10) Handling of the intellectual property right
   11) The methods for publication of the results
   12) Fundings of the study, possible conflicts of interest
   13) Preservation of the specimen, and keeping periods
   14) Contact address, person
   15) With or without compensation, in case of the occurrence of adverse effects
   17.3 Approval of the institute
   After obtained the approval of the Research Ethics Committee of the Osaka University Hospital, the study can be started.
   17.4 Protection of personal information
   We will do maximum effort to protect the personal data of the participants, according to the law of personal information. We use identifying code instead of the personal identifier, and prevent identifying the person.
3. Costs of the research
   18.1 Funding and Conflict of interest
   This study is supported by Grants-in-Aid (JSPS #2340036, and JSPS#23592287) from the Ministry of Education, Culture, Sports, Science and Technology, Japan.
   This study is partially supported by the global COE program “Center of Human-friendly Robotics Based on Cognitive Neuroscience.”
   18.2 Budget
   All costs are paid from the research expenses.
   18.3 Compensation for health hazard
   For the treatment of the health hazard, health care insurance is applied. We also take out an insurance for clinical studies to compensate the adverse effects.
4. Utilization and preservation of the data
   Secondary utilization of the data requires another approval of the Research Ethics Committee of the Osaka University Hospital.
5. Publication of the outcomes
   The results will be presented in the scientific meetings, and will be published in a journal. Authors will be determined after discussion.
6. Organization of the research
   21.1 Responsible person
   Satoshi Hagihira,
   Department of Anesthesiology and Intensive Care Medicine,
   Osaka University Graduate School of Medicine
   2-2 Yamadaoka, Suita City, Osaka, 565-0871
   Tel. +81-6(6879)-3133, Fax. +81-6(6879)-3139
   21.2 Head office of the study
   Aya Nakae,
   Department of Anesthesiology and Intensive Care Medicine,
   Osaka University Graduate School of Medicine
   2-2 Yamadaoka, Suita City, Osaka, 565-0871
   Tel. +81-6(6879)-3133, Fax. +81-6(6879)-3139
   21.3 Registration center
   Principal roles
   (1) Acceptance of registration, verification
   (2) Assignment of the registration number, and notification
   (3) Assignment of the code, and notification
   Naoyuki Osaka,
   Department of experimental psychology,
   Kyoto University Graduate School of Letters
   Yoshidahonmachi, Sakyou-ku, Kyoto, 606-8501
   Tel.&Fax. +81-75(753)2810
   21.4 Responsible person for Statistics
   Selection of the study design, setting of the including number of participants, methods of statistical analysis
   Aya Nakae,
   Department of Anesthesiology and Intensive Care Medicine,
   Osaka University Graduate School of Medicine
   2-2 Yamadaoka, Suita City, Osaka, 565-0871
   Tel. +81-6(6879)-3133, Fax. +81-6(6879)-3139
   21.5 Responsible person for data management
   Maintenance of database, making the report formats
   Aya Nakae,
   Department of Anesthesiology and Intensive Care Medicine,
   Osaka University Graduate School of Medicine
   2-2 Yamadaoka, Suita City, Osaka, 565-0871
   Tel. +81-6(6879)-3133, Fax. +81-6(6879)-3139
   21.6 Responsible person for assignment
   Naoyuki Osaka,
   Department of experimental psychology,
   Kyoto University Graduate School of Letters
   Yoshidahonmachi, Sakyou-ku, Kyoto, 606-8501
   Tel.&Fax. +81-75(753)2810
   21.7 Assessment of the Safety
   Lin Maeda,
   Department of Anesthesiology,
   Nishinomiya manucipal, Nishinomiya-Cyuoh Hospital
   8-24 Hayashidacyou, Nishinomiya City, Hyogo, 663-8014
   Tel +81-798(64)15151, Fax +81-798(67)4811
   Kunihiro Nakai,
   Department of Plastic Surgery,
   Sakai municipal, Sakai Hospital
   1-1-1 Minami-Yasuicyou, Sakai City, Osaka, 590-0064
   Tel. +81-72(221)1700
7. References
   1) Inada E. Intelｌectual approach for anesthesia. Nihon Iji Sinpon, 2009
   2) Masui K. Can monitoring prevent awareness during anesthesia? LiSA 2009;16: 1026-31.
   3) Ghoneim MM, Block RI, Haffarnan M, et al. Awareness during anesthesia: risk factors, causes and sequelae: a review of reported cases in the literature. Anesth Analg, 2009;108:527-35.
   4) Isumi Y. Anesthetics and memory. Jp J Clin Anesth. 2008;28:191-202.
   5) Kanbara T, Shingu K. Appropriate depth of anesthesia, depth of anesthesia and its sign. Jp J Clin Anesth. 2003;23:117-25.
   6) Morimoto Y, Yoshikawa S. What is the memory recall after surgery? LiSA 2009;16:1018-21.
   7) Nishikawa K. Anesthesia method to prevent memory recall after surgery. LiSA 2009;16:1022-24.
   8) Kazama T. Monitor for depth of anesthesia. Jp J Clin Anesth. 2000;20:7-11.
   9) Avidan MS, Zhang L, Burnside BA, et al. Anesthesia awareness and the bispectral index. N Engl J Med. 2008;358:1097-1108.
   10) Asukai N. How to manage a patient who previously experienced awareness during anesthesia. LiSA 2009;16;1032-5.
   11) Barakat AR, Sutcliffe N, Schwab M. Effect site concentration during propofol TCI sedation: a comparison of sedation score with two pharmacokinetic models. Anaesthesia, 2007;62:661-6.
   12) Sakaguchi et al., Dental sedation for patients with intellectual disability: a prospective study of manual control versus Bispectral Index-guided target-controlled infusion of propofol. J Clin Anesth 2011;23:636-42.
   13) Miyake W, Oda Y, Ikeda Y, et al., Electroencephalographic response following midazolam-induced general anesthesia: relationship to plasma and effect. J Anesth 2010;24:386-93.
   14) Fukuda S. Effect of anesthetic on memory formation. Igakunoayumi. 2010;234:141-7.
   15) Buffett-Jerrott SE, Stewart SH. Cognitive and sedative effects of benzodiazepine use. Curr Pharm Des, 2002;8:45-58.
   16) Olkkola KT, Ahonen J. Midazolam and other benzodiazepines. Handbook of Experimental Pharmacology, 2008;182:335-60.
   17) Hashimoto K, Okada T, Seo N. Propofol doesn't cause retro-active amnesia. Masui 20087;56:920-4.
   18) Matsuki Y, Ichinohe T, Kaneko Y,: Amnesia for electric dental pulp stimulation and picture recall test under different levels of propofol or midazolam sedation, Acta Anestheciol Scand. 2007;51:16-21.
   19) Roode A, van Gerven AMG, Schoemaker RC, et al. A comparison of the effects of propofol and midazolam memory during two levels of sedation by using target-controlled infusion. Anesth Analg 2000;91:1056-61.
8. Appendix
